# Incidence and Risk Factors of Postpartum Hemorrhage in Cesarean Versus Vaginal Deliveries: A Retrospective Study

**DOI:** 10.7759/cureus.95714

**Published:** 2025-10-29

**Authors:** Hina Sajjad, Nimra Shakeel, Saadia Kanwal, Sehrish Sabir, Sana Nadeem, Hina Arshad

**Affiliations:** 1 Obstetrics and Gynecology, Shalamar Hospital, Lahore, PAK; 2 Obstetrics and Gynecology, Sir Ganga Ram Hospital, Lahore, PAK; 3 Obstetrics and Gynecology, Benazir Bhutto Hospital, Rawalpindi, PAK; 4 Obstetrics and Gynecology, District Head Quarter (DHQ) Teaching Hospital, Gujranwala, PAK

**Keywords:** cesarean delivery, maternal outcomes, postpartum hemorrhage, risk factors, vaginal birth

## Abstract

Background

Postpartum hemorrhage (PPH) remains a leading cause of maternal morbidity and mortality globally. The mode of delivery is a known determinant of hemorrhagic risk, yet comparative data on cesarean versus vaginal deliveries remain context-specific.

Objective

This study aimed to determine the incidence of PPH and evaluate its associated risk factors in cesarean and vaginal births.

Methods

This retrospective study was conducted at Shalamar Hospital, Lahore, Pakistan, from April 2022 to April 2025. A total of 325 postpartum patients were enrolled in the study, comprising women who delivered either by cesarean section or vaginal delivery. Data were collected from medical records using a structured proforma, which included demographic details (age, parity, and gestational age), clinical variables (anemia status, induction of labor, duration of labor, and previous cesarean delivery), and delivery characteristics.

Results

The overall incidence of PPH was 19.4% (n = 63), with a significantly higher rate in cesarean deliveries (26.5%) compared to vaginal deliveries (12.3%) (p < 0.01). Uterine atony was the most frequent cause in both modes. Risk factors significantly associated with PPH included maternal anemia (p < 0.001), prolonged labor (p = 0.02), and labor induction (p = 0.01). Logistic regression identified cesarean delivery (AOR: 2.4, 95% CI: 1.3-4.5), anemia (adjusted odds ratio (AOR): 3.2, 95% CI: 1.7-6.0), and prolonged labor (AOR: 1.9, 95% CI: 1.0-3.4) as independent predictors of PPH.

Conclusion

Cesarean delivery, maternal anemia, and prolonged labor significantly increased the risk of PPH. Preventive strategies focusing on antenatal anemia correction, cautious decision-making regarding cesarean deliveries, and timely labor management may reduce the incidence and severity of postpartum hemorrhage.

## Introduction

Postpartum hemorrhage (PPH) is a serious and potentially life-threatening complication of childbirth and continues to be one of the leading causes of maternal mortality and morbidity worldwide. According to the World Health Organization (WHO), PPH is the loss of 500 ml and above 24 hours following vaginal delivery and 1000 ml following a cesarean section [1]. There are primary PPH (occurring within 24 hours of delivery), secondary PPH (occurring between 24 hours and six weeks of giving birth), and, most often, a fatal outcome is attributed to primary PPH [2]. Even though there are better obstetric and surgical skills, effective uterotonic medications, and uniform response systems, PPH still causes thousands of maternal deaths each year, especially in low-income countries where an immediate reaction is restrained [3]. Incidences of PPH are reported to be between 2 and 6% in vaginal births and occasionally up to 10% in cesarean deliveries, but real rates depend on the population, definitions of PPH, and measuring techniques. In Pakistan, more than one-third of maternal causes of death are attributed to hemorrhage, and it is, therefore, pivotal to examine the factors that expose a person to the condition and adopt prevention measures [4]. The amount of blood lost alone is not dangerous, but the rate at which they are lost, as well as how well the body tries to compensate. Some risk factors have been identified in the development of PPH.

Among them are uterine atony (it can be seen as a reason most frequently), placental tissue remaining, injuries of the genital tract, coagulopathies, prolonged or forceful labor, multiple pregnancies, polyhydramnios, high parity and having had a previous cesarean birth and use of drugs to induce labor like oxytocin or misoprostol [5]. Old age, maternal age, anemia, and obesity are also risk factors for becoming vulnerable to blood loss complications. Nevertheless, it is important to conclude that PPH may happen to women without any possible risk factor. Thus, all births must take place in a setting with emergency preparedness, despite the perceived risk level [6]. The mode of delivery plays a significant role in influencing the risk and severity of PPH. Cesarean section, though often life-saving, is inherently associated with greater blood loss compared to vaginal delivery [7]. Anesthesia effects on uterine tone, surgical trauma, prolonged procedure duration, postoperative infection, and other factors can all increase bleeding [8]. Contrarily, however, hemorrhage is also linked to vaginal deliveries, particularly those that require instrumentation (forceps or vacuum extraction), perineal tears, or manual placental removal. The difficulty lies in determining whether PPH is brought on by the mode of delivery itself or by preexisting conditions that made that mode of delivery necessary [9]. The rising rates of cesarean sections globally have sparked debate regarding their appropriateness and long-term consequences [10]. While cesareans are vital in many obstetric emergencies, their overuse in non-emergent situations exposes women to unnecessary risks, including PPH, wound infections, thromboembolism, and delayed recovery. On the other hand, hemorrhagic complications can also occur when high-risk vaginal deliveries are not properly monitored or managed. So, the question is not just which method of delivery is safer, but also in what clinical contexts and with what precautions each method is used [11].

Objective

This study aimed to determine the incidence of PPH and evaluate its associated risk factors in cesarean and vaginal births.

## Materials and methods

Methodology

This retrospective study was conducted at Shalamar Hospital, Lahore, Pakistan, from April 2022 to April 2025. A total of 325 postpartum patients were enrolled in the study, comprising women who delivered either by cesarean section or vaginal delivery. The sample size was calculated using the WHO sample size calculator, with a 95% confidence level, 5% margin of error, and estimated prevalence of postpartum hemorrhage based on prior literature [12]. Non-probability consecutive sampling was used to recruit all eligible participants during the study period.

Inclusion and exclusion criteria

Women aged 18-45 years who delivered either via cesarean section or vaginal delivery at the study site were included, provided they had a gestational age of ≥37 weeks, singleton pregnancies, and gave written informed consent. Patients were excluded if they had known coagulation disorders or thrombocytopenia, a history of uterine fibroids or previous uterine surgeries (excluding cesarean section), multiple gestation pregnancies, stillbirths or intrauterine fetal deaths, or were referrals from peripheral centers after delivery.

Data collection

Data were retrospectively extracted from hospital records using a structured proforma. Information retrieved included demographic details (age, parity, and gestational age), clinical characteristics (anemia status, induction of labor, duration of labor, and previous cesarean delivery), and delivery-related factors. The mode of delivery was noted, and estimated blood loss was documented as recorded in patient files, based on calibrated collection containers and visual estimation performed by trained obstetric staff. Management strategies for postpartum hemorrhage, such as the use of uterotonics, surgical interventions, or blood transfusion, were also reviewed and recorded. The data abstraction process was supervised by senior investigators to minimize bias, and missing data were handled through case-wise exclusion where variables were incomplete.

Statistical analysis

Data were analyzed using the Statistical Package for Social Sciences (SPSS) version 26 (IBM Corp., Armonk, New York, USA). Descriptive statistics (mean ± standard deviation for continuous variables; frequencies and percentages for categorical variables) were used to summarize participant characteristics. The chi-square test was applied to assess the association between delivery mode and incidence of PPH. Logistic regression analysis was performed to identify independent risk factors for PPH. A p-value of <0.05 was considered statistically significant.

## Results

The study included 325 women, with an almost equal distribution of cesarean (49.8%) and vaginal deliveries (50.2%). The mean maternal age was 28.7 years. Anemia (Hb <10 g/dL) was noted in 37.5% of participants, while 31.4% had multiparity. Labor induction or augmentation was used in 30.2% of cases, and prolonged labor occurred in 23.4%, indicating a notable prevalence of clinical risk factors for postpartum complications (Table 1).

**Table 1:** Demographic and clinical characteristics (n = 325). Data are represented as mean ± SD or n (%).

Variable	Value
Total patients	325
Cesarean deliveries	162 (49.8%)
Vaginal deliveries	163 (50.2%)
Mean age (years)	28.7 ± 4.9
Anemia (Hb <10 g/dL)	122 (37.5%)
Multiparity (≥3 deliveries)	102 (31.4%)
Labor induction/augmentation	98 (30.2%)
Prolonged labor (>12 h)	76 (23.4%)

Postpartum hemorrhage (PPH) occurred in 19.4% of total deliveries. The incidence was significantly higher in cesarean deliveries (26.5%) compared to vaginal deliveries (12.3%), with a p-value <0.01 (χ² = 8.36). This indicates that cesarean section is significantly associated with a higher risk of PPH, warranting targeted monitoring in these cases (Table 2).

**Table 2:** Incidence of postpartum hemorrhage. Data are represented as n (%). Comparison between cesarean and vaginal delivery was performed using the chi-square (χ²) test. Significance level: p < 0.05. PPH: postpartum hemorrhage.

Delivery mode	PPH cases (n, %)	p-value	Test statistic
Cesarean (n = 162)	43 (26.5%)	<0.01	χ² = 8.36
Vaginal (n = 163)	20 (12.3%)		
Total (n = 325)	63 (19.4%)		

Among cesarean-related PPH cases, uterine atony was the leading cause (55.8%), followed by surgical bleeding (25.6%) and placenta previa (11.6%). Vaginal PPH was most commonly due to perineal trauma (45.0%), followed by uterine atony (35.0%) and retained placenta (20.0%). These differences underscore the distinct pathophysiology of PPH depending on the mode of delivery (Table 3).

**Table 3:** Causes of PPH by delivery mode. Data are represented as n (%). PPH: postpartum hemorrhage.

Cause of PPH	Cesarean (n = 43)	Vaginal (n = 20)
Uterine atony	24 (55.8%)	7 (35.0%)
Surgical bleeding	11 (25.6%)	–
Placenta previa	5 (11.6%)	–
Perineal trauma	–	9 (45.0%)
Retained placenta	–	4 (20.0%)

Anemia was present in 43 out of 63 PPH cases (68.3%) compared to 80 out of 262 non-PPH cases (30.5%), with a highly significant p-value <0.001 and χ² = 30.56. Multiparity was seen in 26 (41.3%) PPH cases versus 76 (29.0%) non-PPH (p = 0.04, χ² = 4.09). Labor induction was noted in 27 (42.9%) PPH cases vs. 71 (27.1%) non-PPH (p = 0.01, χ² = 6.35), and prolonged labor occurred in 21 (33.3%) PPH cases vs. 55 (21.0%) non-PPH (p = 0.02, χ² = 5.25). These trends confirm significant associations between these factors and PPH (Table 4).

**Table 4:** Comparison of risk factors in PPH vs no PPH. Data shown as mean ± SD or n (%). Associations were assessed using the chi-square (χ²) test. Significance level: p < 0.05. PPH: postpartum hemorrhage.

Risk factor	PPH group (n = 63)	No PPH group (n = 262)	p-value	Test statistic
Anemia	43 (68.3%)	80 (30.5%)	<0.001	χ² = 30.56
Multiparity	26 (41.3%)	76 (29.0%)	0.04	χ² = 4.09
Labor induction	27 (42.9%)	71 (27.1%)	0.01	χ² = 6.35
Prolonged labor	21 (33.3%)	55 (21.0%)	0.02	χ² = 5.25

Adjusted odds ratios revealed that cesarean delivery significantly increased the odds of PPH (OR = 2.4, 95% CI: 1.3-4.5, p = 0.005). Anemia was a strong predictor with an adjusted OR of 3.2 (95% CI: 1.7-6.0, p < 0.001), and prolonged labor also showed significance (OR = 1.9, 95% CI: 1.0-3.4, p = 0.04). Multiparity had a non-significant OR of 1.3 (95% CI: 0.7-2.4, p = 0.29), and maternal age was not associated with PPH risk (OR = 1.1, 95% CI: 0.9-1.3, p = 0.23) (Table 5).

**Table 5:** Logistic regression analysis of risk factors for PPH. ORs with 95% CIs are reported. Multivariable analysis performed using binary logistic regression. The significance level is set at p < 0.05. PPH: postpartum hemorrhage.

Variable	Adjusted OR (95% CI)	p-value
Cesarean delivery	2.4 (1.3–4.5)	0.005
Anemia	3.2 (1.7–6.0)	<0.001
Prolonged labor	1.9 (1.0–3.4)	0.04
Multiparity	1.3 (0.7–2.4)	0.29
Maternal age	1.1 (0.9–1.3)	0.23

## Discussion

This study aimed to determine the incidence and associated risk factors of postpartum hemorrhage (PPH) in cesarean versus vaginal deliveries among 325 postpartum women. The overall incidence of PPH was 19.4%, with a significantly higher rate in cesarean deliveries (26.5%) compared to vaginal deliveries (12.3%) (p < 0.01). These findings are consistent with existing literature, which has frequently reported a higher risk of PPH in operative deliveries due to surgical trauma, uterine atony, and prolonged exposure to anesthetic agents that may impair uterine contractility. Uterine atony was the most common cause of PPH in both delivery modes, but it was particularly prevalent in cesarean births (55.8%). Among vaginal deliveries, perineal trauma (45.0%) and retained placental fragments (20.0%) were significant contributors [13]. These findings support prior observations that while uterine atony is the most common cause overall, delivery-specific causes like surgical site bleeding or soft tissue trauma also play critical roles. Identification of delivery-specific risk profiles is essential for implementing appropriate preventive strategies [14].

This paper identified numerous clinical factors that had significant association with PPH. Hemorrhage incidents in 68.3% of the PPH group versus 30.5% of the non-PPH group were strongly associated with anemia, which was detected in 37.5% of all participants (p < 0.001). This is consistent with findings, which have shown that maternal anemia impairs the toleration of blood loss in the body and also leads to poor outcomes. On the same note, a more extensive labor and induction of labor were also correlated with the chances of acquiring PPH, which is probably because of uterine muscle fatigue, or during processes of exposure to excessive amounts of uterotonics, which have the ability to interfere with the effective contraction of the uterus after delivery [15]. The logistic regression analysis further ascertained cesarean delivery, maternal anemia, and prolonged labor as independent predictors of PPH. Although it is indicated that multiparity and maternal age were significant in univariate analysis, these factors were not independent predictors after confounding factors were considered [16]. The result indicates a multifactorial etiology of PPH and points to the relevance of taking into consideration clinical interactions when assessing risks. Notably, in this study, Tariq et al. emphasize that cesarean delivery not only exposes people to increased risks of hemorrhage but also worsens them. Almost 40% of women with PPH after cesarean needed blood transfusion, and some of them needed surgical treatment like B-Lynch suture and uterine artery ligation as well [17]. Comparatively, all the PPH cases were not subjected to surgical treatment during vaginal delivery. It also confirms the idea that cesarean-associated hemorrhages are more complicated and challenging to deal with, which once again requires precision in the surgical technique and early preparation of advanced management to apply when operating a delivery [18].

The findings of this study have important clinical implications. Firstly, routine antenatal screening and correction of anemia should be emphasized as a modifiable risk factor. Secondly, cautious decision-making regarding cesarean delivery is warranted, especially in borderline cases where vaginal delivery may be safe. Thirdly, active management of the third stage of labor (AMTSL) and the availability of uterotonics, trained staff, and emergency blood products are critical components of PPH prevention strategies, particularly in cesarean settings.

Limitations of this study include its single-center, retrospective design, which may limit generalizability and introduce potential selection bias due to non-random sampling. The reliance on visual estimation for blood loss measurement may have led to observer variability despite standardized assessment protocols. The study also did not assess long-term maternal outcomes such as delayed hemorrhage, anemia recovery, or psychological impact. Although variables such as emergency versus elective cesarean section, indication for surgery, intraoperative complications, surgeon experience, and uterotonic dosage are recognized as important confounders influencing postpartum hemorrhage risk, these were not adjusted for due to incomplete documentation. As a retrospective study, causal relationships between identified risk factors and postpartum hemorrhage cannot be firmly established. Future prospective multicenter studies with comprehensive data collection are warranted to address these limitations and enhance external validity.

## Conclusions

Postpartum hemorrhage remains a major cause of maternal morbidity and mortality, with its incidence significantly influenced by the mode of delivery. In this retrospective study, cesarean deliveries were associated with a higher risk of PPH compared to vaginal births, while factors such as anemia, prolonged labor, and previous cesarean section were identified as significant contributors. These findings highlight the importance of antenatal correction of anemia, vigilant intrapartum monitoring, and preparedness for timely management in high-risk cases. The findings of this study also underscore the need for future prospective, multicenter research to develop predictive models for postpartum hemorrhage risk and to evaluate preventive interventions. Improved documentation and adjustment for confounding factors, such as surgical indication and intraoperative events, will further strengthen the evidence base in this domain.
